# Among Those Who Hinder Progress

**Published:** 1900-06

**Authors:** 


### EDITORIALS.

### AMONG THOSE WHO HINDER PROGRESS.

It is astounding to read in various livestock and agricultural journals such sublime attitudes as many of them are willing to assume in view of the great advances being made in the control of tuberculosis. Rather than to adopt such measures as are now known to be necessary for its control; rather than to adopt some plan leading to its eradication, and rather than to place restraining measures to govern its spread, they are playing the rôle of injured innocence, and inasmuch as they have not contracted the disease themselves they are willing to go on and take the risk of the same rather than to support measures for prevention. These are the same people who objected to the use of petroleum for lighting, then gas, later on electricity, as superior to tallow dips, but they have come to pass, and the latter relegated to obscurity. They objected to steam roads because they displaced omnibus lines and prairie schooners; they fought trolley lines because they displaced horses—too menial and low a place for the noble horse—and they will go on objecting and obstructing until they are dead and gone. They live not to learn, they never forget anything. They still want to have in their homes consumptives without any precautionary methods or restraint. They want tuberculous patients to continue to spit on the floors of public vehicles and in public rooms. They still want to eat tuberculous meat, and rear their children on milk from tuberculous herds, and then charge Providence with bringing down on their heads sickness and death, the result of criminal negligence, and are wont to obstruct every measure and safeguard to keep them from their own folly and the exposure of others to like dangers. They prate about the destruction of 2 per cent. of the bovine species of our land, when evidence is on every hand throughout the Eastern section of our country where its ravages have wiped out entire herds, due to its contagious and infectious character. They are willing to further expose our practically free Western herds rather than join in protective measures for their salvation. That some particular friend or farmer may keep a polluted herd, they will object to measures that shall safeguard his neighbors from similar experiences and losses.

The time has come when these objectors and obstructionists must be set aside and sent to the rear that we may enjoy the beneficent results that are sure to follow the now well defined lines of control work in dealing with the "white plague."

